# Assessing the impact of an evidence- and consensus-based guideline for controlling SARS-CoV-2 transmission in German schools on decision-making processes: a multi-component qualitative analysis

**DOI:** 10.1186/s12961-023-01072-9

**Published:** 2023-12-19

**Authors:** Katharina Wabnitz, Mike Rueb, Eva A. Rehfuess, Brigitte Strahwald, Lisa M. Pfadenhauer

**Affiliations:** 1 Institute for Medical Information Processing, Biometry and Epidemiology (IBE), Chair of Public Health and Health Services Research, Ludwig-Maximilians-Universität München, Elisabeth-Winterhalter-Weg 6, 81377 Munich, Germany; 2 Pettenkofer School of Public Health, Munich, Germany

**Keywords:** Public health guideline, Emergency guideline, Impact assessment, Evidence-informed decision-making, COVID-19, Evidence-based policy-making

## Abstract

**Introduction:**

During the COVID-19 pandemic, decision-making on measures to reduce or prevent transmission of SARS-CoV-2 in schools was rendered difficult by a rapidly evolving and uncertain evidence base regarding their effectiveness and unintended consequences. To support decision-makers, an interdisciplinary panel of scientific experts, public health and school authorities as well as those directly affected by school measures, was convened in an unprecedented effort to develop an evidence- and consensus-based public health guideline for German schools. This study sought to assess whether and how this guideline impacted decision-making processes.

**Methods:**

This study comprised three components: (1) we sent inquiries according to the Freedom of Information Acts of each Federal State to ministries of education, family, and health. (2) We conducted semi-structured interviews with individuals involved in decision-making regarding school measures in two Federal States, and (3) we undertook semi-structured interviews with members of the guideline panel. The content of response letters in component 1 was analysed descriptively; data for components 2 and 3 were analysed using deductive-inductive thematic qualitative content analysis according to Kuckartz.

**Results:**

Responses to the Freedom of Information Act inquiries showed that the guideline was recognised as a relevant source of information by ministries of education in nine out of 16 Federal States and used as a reference to check existing directives for school measures in five Federal States. All participants (20 interviews) emphasised the value of the guideline given its evidence- and consensus-based development process but also noted limitations in its usability and usefulness, e.g., lack of context-specificity. It was consulted by participants who advised policy-makers (5 interviews) alongside other sources of evidence. Overall, perceptions regarding the guideline’s impact were mixed.

**Conclusions:**

Our findings suggest that the guideline was relatively well-known in Federal States’ decision-making bodies and that it was considered alongside other forms of evidence in some of these. We suggest that further research to evaluate the impact of public health guidelines on (political) decision-making is warranted. Guideline development processes may need to be adapted to account for the realities of decision-making during public health emergencies and beyond.

## Introduction

Decision-making regarding public health and social measures (PHSM) during the coronavirus disease 2019 (COVID-19) pandemic had to take place in “greatly compressed timeframes, and in situations with complex, intersecting social, economic, and political pressures” [1]. PHSM aim to reduce the risk of transmission and, consequently, to reduce the burden on health systems, economies, and societies [2]. There is an expectation that scientific evidence for effectiveness should be considered in (public) health policy decisions [3, 4]. A rapidly evolving, sometimes conflicting, and partly unscrutinised evidence-base regarding the effectiveness of PHSM posed significant difficulties [5]. In addition, decision-makers had to factor in (unintended) effects beyond health outcomes.

Schools are illustrative of these challenges: school closures were among the most disruptive PHSM in the early stages of the pandemic [6]. Supporting evidence of effectiveness for school closures was almost entirely derived from previous research on seasonal influenza control; the available evidence on the effects of school closures on coronavirus control, including COVID-19, was scarce and inconclusive [7]. Concurrently, evidence for the potential negative impacts of school closures on students’ health and psychosocial wellbeing, educational attainment, parental productivity and income, and the health care system, notably through absenteeism of female caregivers, was available from previous outbreaks [8–10]. In light of this, and with evidence emerging that the impacts of COVID-19 on younger age groups were less severe and that school-based outbreaks played a minor role in community transmission [11], the goal to keep schools open gained political traction at the international level [12]. Similarly, Germany’s Federal States’ ministers of education passed a resolution in October 2020, affirming that face-to-face teaching should have priority in all political decisions regarding COVID-19 infection prevention and control [13].

Measures to keep schools open safely by preventing and controlling transmission of severe acute respiratory syndrome corona virus 2 (SARS-CoV-2) (ff. school measures, excl. school closures) include interventions to reduce the opportunity for contacts such as reducing the number of students and staff; to make contacts safer such as mask mandates; and surveillance and response measures such as testing [14]. Their effects depend on community transmission levels and on the implementation of other PHSM in the community, among others [15].

In Germany, pandemic management, i.e., the development and implementation of legal directives for PHSM, is a legislative competence of the 16 Federal States which resulted in divergent levels of restriction, including in schools, across States [16]. Policy advice on PHSM and the potentially negative societal impacts of PHSM has been sought by politicians at the national and Federal State levels, including from individual experts, institutions such as the Robert Koch Institute (RKI, German National Public Health Institute), ad hoc established expert committees and existing bodies such as the German National Academy of Sciences Leopoldina [17]. Data from 2020 showed that newly established expert committees were dominated by biomedical expertise and the results and procedures of their advisory activities were mostly intransparent [18]. After the first pandemic-related school closures in Germany in spring 2020, a second partial lockdown was implemented in Germany in autumn 2020, and included school closures from December 2020. Against this backdrop, an interdisciplinary panel of scientific experts and stakeholders representing students, parents and teachers (ff. school family) as well as public health authorities, was convened to develop an evidence- and consensus-based guideline (ff. S3-guideline) for school measures.^1^ This process was new in Germany in many respects and was evaluated in a separate study [19]. Established procedures for the development of clinical guidelines by the Association of the Scientific Medical Societies (AWMF, Arbeitsgemeinschaft für Medizinisch-Wissenschaftliche Fachgesellschaften) were adapted to the development of this guideline. Specifically, the available evidence was rapidly and systematically searched and formal consensus-building procedures were followed, using an online voting tool to arrive at recommendations. Furthermore, the WHO-INTEGRATE Evidence-to-Decision framework (EtD) was applied—to our knowledge for the first time during a guideline development process in Germany [20].

The process resulted in the publication of the first short version of the ‘S3-guideline for the prevention and control of SARS-CoV-2 transmission in schools’ in February 2021 [21]. The guideline was presented at a press conference by the then Federal Minister for Education and Research on 8th February 2021 and described in the Ministry’s official media communication as “an important contribution by science in pandemic times” [22]. Subsequently refined short and a long versions, including detailed evidence summaries, were published in November 2021 [21]. The development of this rapid guideline required substantial human resources and was challenging due to a lack of valid and reliable evidence and the short time frame for its development, among others [19]. In light of these circumstances, it is crucial to understand to what extent this guideline as a tool for science-based policy advice during the COVID-19 pandemic had an impact on political and practical decision-making regarding school measures in Germany. The insights gained may inform strategies for effective evidence-to-policy strategies during future public health crises.

### Aim

This study sought to assess whether and in what way the ‘S3-guideline for the prevention and control of SARS-CoV-2 transmission in schools’ impacted decision-making processes regarding school measures in Germany. Impact was defined as knowledge about and use of the S3-guideline by decision-makers in policy and practice. We sought to examine impact from the perspective of those involved in decision-making on the Federal State level as well as from the perspective of guideline panel members.

## Methods

### Design

We adopted a multi-component approach to examine the guideline’s impact from different angles, consisting of *inquiries* with relevant ministries according to the Freedom of Information Acts (FoIA, German *Informationsfreiheitsgesetze*) of all German Federal States (component 1), *semi-structured interviews* with individuals involved in informing or making decisions in two Federal States (component 2) and *semi-structured interviews* with members of the guideline panel (component 3) (see Table 1). For component 2, we chose to recruit within Bavaria (the second most densely populated and a comparably wealthy area state) and Bremen (a small, less well-off city-state) to capture similarities and differences between these different settings. Qualitative research serves to learn from the perspectives of individuals involved in or informed about a phenomenon of interest, here the impact of the S3-guideline. Under normal circumstances, we would have primarily relied on component 2 to address our research objectives. During pandemic circumstances, we expected a low response rate, given enormous time pressures on decision-makers. We therefore decided to complement component 2 with inquiries according to the FoIA, and with interviews with members of the guideline panel. We expected this component to yield thick data, given the insights held by panel members with regards to political and practical decision-making processes affecting schools and the fact that many panel members were directly consulted in such processes.

**Table 1:** Overview of study components

	Component 1	Component 2	Component 3
Sampling frame	16 Federal State ministries of education, family and health	Individuals involved in decision-making regarding school measures in Bavaria and Bremen	Guideline panel members
Data collection	Inquiries via e-mail according to FoIA of each Federal State	Semi-structured interviews	Semi-structured interviews
Data analysis	Categorisation of responses	Thematic qualitative content analysis according to Kuckartz	Thematic qualitative content analysis according to Kuckartz
Integration	Narrative integration of results	Narrative integration of results	Narrative integration of results

Illustrative quotes were extracted from data in all three components, translated from German to English (KW) and checked by a second author (MR). The paper follows the consolidated criteria for reporting qualitative research (COREQ) checklist (Appendix 5) [23]. Findings across the three components were integrated narratively.

### Data management and consent

All interview data were securely stored on an encrypted and password-protected device and anonymised using a combination of letters and numbers and replacing any mention of institutions or locations by neutral descriptions. Written informed consent to participate in the study and for the data to be included in prospective publications was provided by all participants prior to data collection.

### Component 1: Inquiries according to Freedom of Information Acts

#### Data collection

Using the online portal FragDenStaat [24], which is run by the not-for-profit Open Knowledge Foundation Deutschland e.V. [25], or publicly available contact details, we sent inquiries according to the FoIA of each Federal State to the ministries of education, family, and health (or the respective departments in case these were part of the same ministry) between 10 and 12th April 2021. These inquiries contained four main questions:

- Is the guideline known in the ministry?
- Was the guideline discussed in the ministry?
- Was the guideline considered for decisions regarding school measures in the ministry or decisions that the ministry was involved in?
- Did the guideline influence information, recommendations, or requirements for schools issued by the ministry?

Main questions were supplemented with more detailed questions (see Appendix 1).

#### Data analysis

The content of all response letters and specifically answers to the four main questions were analysed by two authors (KW, MR) who independently read and categorised data from the response letters into four categories (Yes/No/No answer/Unclear). Any discrepancies in interpretation were resolved through discussion. Results from this component were expected to provide initial insights regarding whether and how the guideline had an impact on decision-making regarding school measures.

### Component 2: Semi-structured interviews with individuals involved in informing or making decisions

#### Sampling and recruitment

Initially, gatekeepers, i.e., individuals within the professional networks of the authors with affiliations to institutions in Bavaria and Bremen and likely involved in decision-making regarding school measures, were identified. Gatekeepers were contacted via e-mail with a request to forward invitations and study information sheets to individuals within their institutions who would likely meet the following study inclusion criterion, i.e. to have been involved in institutional decision-making processes regarding infection prevention and control measures in schools when the short version of the S3-guideline was published on 8th February 2021, and thereafter. Subsequently, we also asked interview participants to suggest further potential interviewees (snowballing) [26].

#### Data collection

Semi-structured interviews were conducted by two researchers (KW, MR) in German from 22nd December 2021 to 24th March 2022 based on a semi-structured interview guide (see Appendix 2). Interviews took place through a web-based video-conferencing tool or by telephone, respecting the preferences of individual participants. Questions covered the following topics: (a) processes of decision-making regarding school measures, (b) sources and channels of scientific and other information, and (c) the S3-guideline’s impact on decision-making processes regarding school measures. Processes of decision-making were only made a subject of discussion in this component to contextualise the data on the guideline’s impact. We also investigated participants’ understandings of evidence and expertise (not reported in this article). Interviews were recorded using a linear pulse-code modulation (PCM) recorder (OLYMPUS LS-P1). The two researchers independently wrote memos following each interview.

#### Data analysis

Audiotranskription, an external transcription service complying with all necessary EU data protection requirements, was commissioned to transcribe all interviews [27]. Audio files were destroyed after transcription. We provided all participants with their anonymised transcript (member check) and resolved any requests for corrections and further anonymisation. A process of reflecting about the researchers’ positionality accompanied data collection, analysis, and write-up. Thematic qualitative content analysis according to Kuckartz was used to analyse the data. This entails applying deductive main categories to the data and then developing inductive categories within those main categories [28]. The explicit use of deductive and inductive elements allowed clustering of the data according to specific aspects of interest while additional themes could also be elicited. The following steps were undertaken: (1) familiarisation with the data (KW); (2) deductive development of main thematic categories based on questions in the interview topic guides (KW); (3) clustering of all data in those main categories (KW); (4) inductive development of sub-categories within each main category and further clustering of data in those categories (KW, with intra-coder reliability established through various rounds of applying the sub-categories to the data); (5) analysis of the thus-structured content within and across main categories (KW with input from all other authors). The qualitative analysis software MAXQDA was used for data management and analysis [29]. For the full coding system, see Appendix 3.

### Component 3: Semi-structured interviews with guideline panel members

The methods used here are largely similar to the methods used in component 2. Below, we briefly describe those methodological steps that differed from the methods described and applied in component 2.

#### Sampling and recruitment

Recruitment of and interviews with guideline panel members took place in the context of a separate study on the strengths and weaknesses of the guideline development process [19]. All guideline panel members were eligible for inclusion in the study. To achieve representation of the different stakeholders within the panel, we assigned all panel members to five groups (scientists, public health practitioners, members of the guideline secretariat, members of the school family, observers) and recruited purposively within these groups.

#### Data collection

Semi-structured interviews were conducted by two researchers (KW, MR) in German from 12th November to 22nd December 2021 using an interview guide (see Appendix 2) [19]. Questions were related to the S3-guideline’s impact on decision-making processes regarding school measures.

### Data analysis

After transcription and an anonymised member check, thematic qualitative content analysis according to Kuckartz was used to analyse the data, following the same steps as described under component 2 [28]. For the full coding system, see Appendix 3.

## Results

We sent 38 inquiries according to each Federal States’ FoIA to the ministry responsible for education, health, and family of each Federal State, respectively. In component 2, a total of five interviews were conducted, including four individuals from Bavaria and one from Bremen (see Table 2). None of them had a political mandate (i.e., were making decisions); however, they were involved in processes of informing and implementing political decisions. In component 3, 15 interviews were conducted with members of the guideline panel, representing the perspectives of all involved stakeholders and the guideline secretariat (see Table 3).

**Table 2:** Study characteristics of component 2 participants* and interview duration

Sample	ID	Group	Duration of interview in minutes
Decision-making	A1	Local health authority	33
Decision-making	A2	Federal State health authority	26
Decision-making	A3	Local health authority	30
Decision-making	A4	Federal State health authority	42
Decision-making	A5	Federal State Ministry of Education	32
Total	5		163

*No further characteristics provided to maintain confidentiality

**Table 3:** Study characteristics of component 3 participants* and interview duration

Sample	ID	Group	Duration of interview in minutes
Guideline panel	B1	Guideline secretariat	40
Guideline panel	B2	Guideline secretariat	43
Guideline panel	B3	Guideline secretariat	41
Guideline panel	B4	Scientist	32
Guideline panel	B5	Scientist	41
Guideline panel	B6	Scientist	26
Guideline panel	B7	Guideline secretariat	50
Guideline panel	B8	Scientist	54
Guideline panel	B9	Public health practitioner	56
Guideline panel	B10	Public health practitioner	40
Guideline panel	B11	School family	29
Guideline panel	B12	School family	46
Guideline panel	B13	School family	34
Guideline panel	B14	School family	27
Guideline panel	B15	Observer	33
Total	15		592

*No further characteristics provided to maintain confidentiality

In the following, we first present our findings related to the processes of decision-making on school measures with individuals involved in decision-making in Bavaria and Bremen. This provides information regarding the context into which the guideline was introduced. We then describe our findings regarding the impact of the guideline, derived from the FoIA inquiries and all interviews.

### Processes of decision-making regarding school measures

*Hierarchies in the development and implementation of decisions* The data that provides the foundation for this category exclusively stems from interviews with individuals involved in decision-making. Recommendations or proposals for measures had to be reviewed across multiple concerned ministries, at all levels of administration up to the level of the Minister or Head of Department in Bavaria.

*„There is first the officer level […] Then there is the unit level, head of unit level. That's the next level that has to agree, […] the next level is the head of department. Above that comes the head of office. And above that comes the minister. […] And then there are always the legal departments, which also look at it. […] And it is often the case that one department is responsible for schools but another is responsible for infection control. And that has to be integrated […] sometimes […] you make a compromise and then it goes to the next level and they reject it and then you start all over again.“* (A2, Federal State health authority)

Binding regulations for local health authorities that were responsible for implementing measures were issued from the Ministries of Health and Education in Bavaria.

*„The for us rather binding specifications come from the Ministry of Health and the Ministry of Education […] [which] apply to all health authorities.“* (A3, local health authority)

In both Federal States, final decisions were described to have been made by the Federal State governments.

„*This was decided upon by the [Federal State government].*“ (A5, Federal State ministry of education)

*„At the Federal State level, decisions were taken and voted upon by the [Federal State government].“* (A1, local health authority)

This participant from a local health authority described that even though their institution was an implementing body that was bound to follow governmental directives, they would have some leeway in decision-making.

*„Basically, we are acting within the scope of the [federal] Protection against Infection Act. And apart from the pandemic, decisions are always made on a case-by-case basis. And as a doctor, you […] always have the right to decide differently in justified cases. […] this pandemic is the first time that specifications have been issued for infection control.“* (A3, local health authority)

However, another participant from the same local health authority pointed out that they had to seek governmental authorisation for their decisions whenever the pandemic regulations contained implementation flexibilities, which was only the case during certain periods of the pandemic.

It was also pointed out that recommendations by the RKI or resolutions at the national level sometimes prevented more stringent measures to be implemented at the Federal State level. This participant mentioned political decision-makers’ legislative powers to have been unusually large in pandemic decision-making: “*[Bavaria] ultimately decided […] and then put it to vote which was not difficult given the majority ratio. […] For some people the democratic process was [quite thin].“ (A1, local health authority).*

*Sources and channels of information and advice for decision-makers* Information and advice was proactively sought by decision-makers, e.g., by commissioning Federal State health authorities or research institutions to offer technical advice or by consulting with individual experts.

*„You get the expertise from where it is in the Federal State […] there are things that politicians commission directly. […] And then, above all, there are expert hearings, some of which took place on extremely short-notice, especially in the case of amendments.“* (A1, local health authority)

This participant said that „*they don’t look at studies in the Ministry.*“ (A2, Federal State health authority) and it was further explained that their role was to make evidence-based suggestions for regulatory decisions. These decisions would, however, not always reflect their advice.

*„My task was to suggest […] what could be changed, included, improved, and the decision as to what was ultimately, let's say, actually implemented or actually put in writing, was not made by me […] I advised and suggested, and this was partly adopted of course, and partly the ministries decided differently.“* (A4, Federal State health authority)

The same participant explained that they would sometimes be involved in discussions at the ministerial level, e.g., in video conferences, or provide written feedback on draft regulations but also implied that they were not always involved in discussions that led up to decisions.

Interdisciplinary emergency task forces both at the Federal State level as well as within the Ministry of Education were set up in Bremen. These groups also issued recommendations to political decision-makers.

*„A recommendation […] then went *via* the [head of the Ministry] to the [government]. In principle, it went to the state government which then decided. This usually corresponded to the consultations. But not always.“* (A5, Federal State ministry of education)

In Bremen, task forces would base their recommendations on the perspectives of different groups of stakeholders as well as on advice sought from a research institute.

*„Health professionals […] met in the task force. […] The Association of Paediatricians and Adolescent Doctors often had a different view than the health authority. […] And that was always […] a constant balancing act […].“* (A5, Federal State ministry of education)

Participants also mentioned the recommendations issued by the RKI and the resolutions passed at the ministerial conferences of all 16 ministers for education as being important in informing decisions at the Federal State level.

Information was also provided by local actors tasked with implementation, e.g., participants from the local health authority in Bavaria described that they would proactively convey their insights to decision-makers. These were based both on their own observations and on their interpretation of the peer-reviewed evidence.

“*We actually had […] our own findings […] simply from observation very early in this pandemic; from, let's say, epidemiological research in inverted commas, i.e., not systematised, scientific research, but assessment of our data, so to speak. And we have certainly tried to convey these findings to higher levels of government*.” (A3, local health authority)

* Consideration of unintended consequences and further aspects when informing and making decisions* Beyond scientific evidence, consideration of unintended consequences, societal implications as well as the interests of different groups were described to influence processes of informing as well as making decisions. This participant described the challenges with assessing the potentially far-reaching effects of measures taken to control transmission.

*“What was new to me in this situation was of course this collateral damage, in inverted commas. […] usually, I have no reason […] to worry about whether a whole generation of children becomes impaired in their language development.”* (A4, Federal State health authority)

This need to consider further aspects in offering technical advice such as (un)intended consequences and feasibility and acceptability of school measures was evident from statements of all participants. For example, it was mentioned that evidence-based recommendations for infection prevention and control were “*watered down*” (A2, Federal State health authority) by considerations of feasibility, which were nevertheless seen as indispensable for implementation. Within the Bavarian local health authority, unintended consequences of school measures, specifically school closures, on student’s psychosocial wellbeing, were said to have been discussed from an early stage and further aspects, for example legal considerations, were included through in-house consultation with their legal department. Similarly, the tensions between what would be stipulated to prevent and control infections and the unintended consequences or implementation issues that could arise from such measures had to be eased within the political advisory bodies in Bremen.

*„Ultimately, it is of course also the task of the health authority and the health ministry to warn about infections in a pandemic. And to take all sorts of measures to prevent them. But that is not our role […] And this role conflict always had to be balanced. […] And some of the things that were proposed [by the health authority] could not be implemented in practice from our point of view.“* (A5, Federal State ministry of education)

It was also described how further factors featured in the process of political decision-making, such as “*economic, societal factors*” (A1, local health authority), “*political will*” (A3, local health authority), and “*costs and effect*” (A4, Federal State health authority). Interest groups, personal experiences and general risk attitudes were also described to influence the political process of arriving at decisions for school measures.

### Impact of the S3-guideline on processes of decision-making regarding school measures

#### Inquiries according to Freedom of Information Acts

**Fig. 1:**
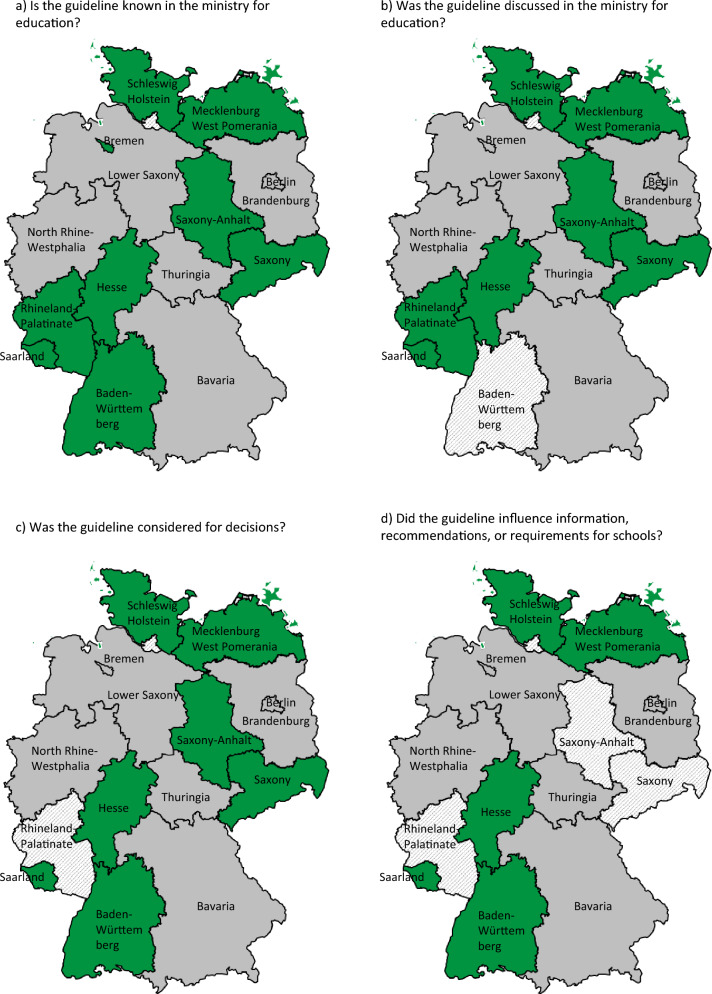
Impact of the S3-guideline on decision-making processes, according to answers to four main questions as part of the FoIA inquiries by Federal States. Green = yes, red = no, grey = no answer, shaded = unclear

All health and family ministries that responded either rejected the inquiry or referred it to the respective Federal State ministry for education. Of the 16 Federal State ministries for education, nine provided answers to all main questions; the remaining seven did not respond (see Fig. 1 for a graphic and Appendix 4 for a tabular overview of results). A range of school measures, legally mandated through directives in each Federal State, was already in place when the guideline was published. The guideline was known to all nine ministries for education that responded. In seven, it was considered in discussions and played a role in decision-making regarding school measures. Notably, five responses explained that the Federal State regulations for schools in place at the time of publication were deliberately checked against the S3-guideline. Of those, two responses outlined specific changes: regulations regarding physical education classes in Mecklenburg-West Pomerania and mask mandates in Saarland were changed in accordance with the guideline’s recommendations.

#### Views of individuals involved in decision-making and guideline panel members

Guideline panel members’ views were similar to those of individuals involved in decision-making. Diverse aspects underpinning categories were mentioned by all participants without a recognisable pattern related to different stakeholder groups.

##### Perceived value of the S3-guideline for pandemic decision-making

Participants described the guideline as a valuable resource, for example, as a systematic assessment of the available evidence and expertise, which would be less prone to bias or subjectivity (as opposed to purely expertise-based recommendations) and could *“potentially simplify or even accelerate decision processes “* (A4, Federal State health authority). It could further be used to justify recommendations, thus providing some accountability from a legal point of view as well as enhancing acceptance according to participants.

*„You can also make it more transparent for a broader public and through this scientific process […] you can significantly increase acceptance within the general population.“* (A4, Federal State health authority)

*„We used it as an argumentation aid […] with politicians, decision-makers. But also, for example, with the [school family] […] And I could point out that […] I’m acting according to a recognised guideline; this renders my own position legally much more solid.“* (A1, local health authority)

*“For my own assessments and my own actions [as an operative part of the pandemic response], the guideline was extremely helpful”* (B4, scientist)

Panel members pointed to the participatory process of developing the guideline and the consideration of unintended consequences in recommendations; in their view, this would render the guideline useful for decision-makers.

“*In general, I think that the guideline was an important attempt to bring together evidence, expert opinion and the perspectives of different stakeholders who are directly affected; at least in the attempt of a fair, transparent, participatory process. And that alone has a very, very high value for me [as] during COVID-19 many decisions were made by individual experts in consultation – as in, the politicians decided of course.”* (B8, scientist)

##### The role of the S3-guideline in political decision-making

Participants’ statements regarding the actual role of the guideline in political decision-making were ambiguous with some stating that they believed it was noticed and considered by decision-makers and others expressing doubts whether this had been the case or saying they were unable to judge this.

*“I do believe that it influenced political decision-makers. That there were some who read up on it […] [that the guideline] has a certain significance, without being able to claim that it shaped politics.”* (A1, local health authority)

*“I personally did not get the impression that it played a big role for decisions. I actually believe that it […] plays a role in the preparation of recommendations. […] I am not so sure whether it constitutes the decisive factor that brings about the decision in the end.”* (A3, local health authority)

“*I actually find it difficult to judge what such a guideline can achieve. I know that the AWMF guidelines on […] medical issues are of course very carefully considered by everyone, but […] I find it difficult with this guideline*.” (B6, scientist)

The following presents the most definite statement which can be considered an outlier compared to the rest of the data underpinning this aspect:

*“I don’t think the guideline has had any influence on the decisions […] I don’t think any decision would have been taken differently, if this guideline had not existed.”* (B7, scientific secretariat)

It was mentioned that recommendations representing a compromise between different perspectives might be less attractive to decision-makers, although the participatory and inclusive process that led to the guideline’s recommendations was perceived as valuable, too. It was reflected on the political traction an evidence- and consensus-based guideline could possibly gain as opposed to direct consultation of individual experts:

*“In politics there is a need to ask experts and to then be able to draw from a certain knowledge eminence. […] in such a guideline process, where you do not say that Professor X has somehow brought this forward, there is less personalisation involved. […] the guideline cannot provide that [as] it is always a consensus. […] we simply have to think about how to communicate and promote such processes and the products of such processes, [emphasising] that they have a similarly high or perhaps even a higher value.”* (B7, scientific secretariat)

“*I think it’s individuals who play a role in getting through to the political decision-making bodies […] [those] who can put forward […] their concern[s] well.*” (A3, Local Health Authority)

Relatedly, one participant highlighted that the guideline might have played a role in political decision-making through those panel members who were also consulted by policy-makers as experts and who might as such have conveyed the essence of the guideline’s recommendations.

*“I [believe] that the guideline was very present in politics at that time and therefore […] played a role in decision-making […] [as] some people who were involved in the guideline ‘s development [have] advised politicians anyway.”* (A4, Federal State health authority)

##### The role of the S3-guideline for decision-making in schools

The data that provides the foundation for this category exclusively stems from interviews with guideline panel members. Regarding the relevance of the guideline for decision-making in schools, views and experiences were mixed with e.g., this panel member clearly stating that *“As headmistress, my ministry of education is the body that issues specifications which I then implement […] I […] don't have much time to read other papers which do not guide my actions either”* (B15, observer). Others were also doubtful about the guideline’s impact on decision-making or implementation of measures, let alone whether the guideline was even widely known at the level of schools.

*“I am not even sure if all school headmasters in Germany know that there is such a guideline. […] So I don't think it had such a huge impact.”* (B14, school family)

One member of the school family stated that for them the guideline helped in conveying a greater feeling of security to their colleagues during times of uncertainty. Another representative of the school family explicitly said they would not refer to the guideline in their daily activities at school. However, this participant said that they “*participated in several conversations […] and there the guideline was definitely also discussed. And those affected in schools on the ground were also present”* (B5, scientist), implying that the guideline was recognised by members of the school family at least in that setting.

##### Perceived limitations to the guideline’s actual impact

Generally, panel members perceived societal recognition of the guideline’s existence and development process as well as its purpose and legitimacy insufficient, notably when compared with other sources of information such as recommendations by the Standing Committee on Vaccination (‘Ständige Impfkommission’, STIKO). This panel member referred to different stakeholders as poorly informed, e.g., the general public not knowing about guidelines:

*“I would say that we were still working underground with the first draft, if you look at it in terms of society as a whole. Yes, it was an elite circle that was working on it. And in society […], S3-guideline means nothing to anybody […] Go out on the street and ask who knows what an S3-guideline is. They usually look at you with their eyes wide open. But if you ask in socially deprived areas what the STIKO is, that has become common sense, people know that.”* (B12, school family)

Another panel member considered political decision-makers to be better informed about the guideline than stakeholders from the school context, i.e., school staff, students, and parents:

*“I have the impression that we were quite good at the level of institutions, political institutions and perhaps also the subordinate authorities. It would also be nice if school headmasters knew the guideline, and I suspect that many do not know that there is such a guideline. […] I believe that the guideline had relatively little impact at the school level, i.e., direct impact.*” (B2, scientific secretariat)

However, this panel member highlighted that “*they did not even know in the ministries of education that it existed […] it took me a relatively long time to convey, even to our ministry in [Federal State], what it actually means “* (B12, school family).

Relatedly, panel members described differences in the reception and uptake of the guideline across different Federal States which are also reflected in the responses to the FoIA inquiries.

*“This has been very different in the individual Federal States. […] [Some] have integrated it immediately into their own guidelines. Others have kept their distance or have not been proactive.”* (B15, observer)

The time lag between the generation of evidence that was included in the guideline process and the publication of the guideline against a backdrop of a rapidly developing body of evidence and changing pandemic circumstances was perceived to be a major limitation to the (potential) impact of the guideline.

*“Certainly important, but no longer up to date. I think that's what you ought to say. It simply lags behind the dynamics and is already out of date the moment it is published.”* (B11, school family)

Hinging on the importance of continuously updating guidelines in light of rapidly changing circumstances, this participant stated: *“If [the RKI] revised their process to include more guideline-like elements, for example taking into account other criteria or obtaining stakeholder statements, they might be able to cope better with such rapid changes. […] institutionalised actors are perhaps more capable of doing this than such external medical guidelines”* (B7, scientific secretariat).

Further limitations to the guideline’s impact mentioned by panel members included its non-binding character. Moreover, *“public perception somehow was that this guideline was a product of the [German Federal Ministry for Education and Research] and was then also politically instrumentalised […] for example, a representative of the Green Party said something against the guideline although, in terms of the process, it is actually exactly what they always advocate for”* (B8, scientist), pointing to the risk for instrumentalization by political or other interest groups. Individuals involved in decision-making said that the guideline was one source among others that were considered to develop recommendations in the emergency task forces and scientific advisory roles that they were part of. Interestingly, one participant insinuated that *“it [the guideline] was often too narrow, [focussed] on the immediate protection of health. In the sense of not contracting the infection. But too little on the consideration of the long-term well-being of children and young people”* (A5, Federal State ministry of education). They said that further information beyond the guideline was needed to build an opinion regarding the unintended consequences of school measures. The guideline was described as providing “guard rails” (A3, local health authority) for decision-making while “’*micro steering’ cannot be informed by such a guideline. It can only provide a frame*” (B12, school family). Therefore, “*it is important to know the limits of guidelines. That […] I say […], What can be implemented in practice now? […] Do I have to say that I can’t implement it like that? And that I would have to justify very well*” (A2, Federal State Health Authority). It could not serve as an aid to implement measures in a context-specific manner or to manage acutely arising issues: *“There are certainly very clear limits to the guideline in acute pandemic events […] but also in science [generally] when acute action is necessary.“* (B4, scientist).

One participant perceived the guideline panel as not sufficiently comprehensive regarding relevant perspectives and gender. Interestingly, it was mentioned that individual experts or studies that were used to inform political decision-making before the guideline was published (some of which were also included there) played a bigger role in informing the decision-making process than the guideline itself.

*“I ask myself, did [the guideline] really play a role? It was informed by opinions […] that were already known before. It was also informed by studies […] Partial opinions that are included in the [guideline] had already been presented to the Ministry […] I think that the [guideline] has been taken note of. However, I believe that individual studies that informed the [guideline] had […] already contributed to the decision-making process beforehand or with a greater weight.”* (A1, local health authority)

## Discussion

### Summary of findings

We sought to assess whether and in what way the ‘S3-guideline for the prevention and control of SARS-CoV-2 transmission in schools’ impacted public health decision-making processes in Germany, using semi-structured interviews and nationwide FoIA inquiries.

Analysis of responses to the FoIA inquiries suggests that the S3-guideline had some impact on political decision-making in those Federal State ministries of education who responded to our inquiries. Ministerial decision-making processes regarding school measures were described as being rather hierarchical in Bavaria and Bremen, with multiple sources of information, including the S3-guideline, being considered. Alongside scientific evidence, further aspects such as unintended consequences of school measures, cost, and feasibility were considered in making decisions.

The views of guideline panel members and individuals involved in decision-making regarding the theoretical value and actual impact of the guideline on decision-making in policy and in schools were mixed. While the participatory and transparent development process and the guideline’s potential to provide accountability were seen as useful, most participants were uncertain regarding its actual role in political and practical decision-making. Several limitations to the impact of the guideline were stated, including lack of widespread awareness of the S3-guideline’s existence and its non-binding character.

### Bridging the “science-policy gap” with a guideline?

#### The role of the guideline in relation to other sources of evidence

A range of school measures, legally mandated through directives in each Federal State, was already in place when the guideline was published. Only two responses to FoIA inquiries indicated that specific changes in individual measures were made according to the guideline. This may suggest that other directives in place were already largely aligned with the guideline’s recommendations. However, a comparative document analysis of directives issued by all Federal States shortly after publication of the guideline found that existing directives were highly heterogenous and showed large discrepancies with the guideline’s recommendations, e.g. regarding air purification [30]. This may suggest diverging sources of evidence or influence playing out in the creation of directives across States. Individuals involved in decision-making elaborated that the S3-guideline did not play a major role in their assessments of the available evidence compared to other sources of information. This was corroborated by guideline panel members’ perceptions regarding the important role of single studies or expert opinion in informing political decision-making. It is also in line with previous studies which found use of guidance issued by NICE or other institutions in the UK to be contingent on aspects of organisational (power and informal knowledge exchange) dynamics as well as local priorities and needs [31–33]. While expert opinion and similar forms of evidence are known to have limited scientific rigour within the scientific community, these are found to be the preferred option to turn to in a variety of decision-making contexts [34–36]. This could also be observed during the pandemic, where consulting with individual experts or pre-existing and newly established expert advisory groups was a common way of integrating scientific knowledge into decision-making in Germany, as well as in many other countries [18, 37, 38].

#### Stakeholder engagement for guideline development, dissemination and implementation

Stakeholder engagement is widely considered an effective strategy for improving the uptake of guideline recommendations [39–42]. Our findings suggest that individual experts who were part of the guideline panel played a critical role in conveying the recommendations included in the guideline to decision-makers. This is in line with other initiatives, where stakeholders are not only engaged during guideline development, but also in the dissemination and implementation of recommendations contained in the guideline [43, 44]. However, degree of representation of stakeholders in this guideline’s panel was questioned by one participant. While this was a singular statement which would require further investigation and triangulation, it triggers questions regarding the extent to which a guideline panel that aims to produce a tool relevant for and used by political decision-makers, should be democratically elected. This could enhance awareness and acceptance among the general population and minimize risks of “issue bias”, i.e. skews in policy-making towards issues with seemingly straight-forward solutions or delays in decision-making on scientifically contentious topics [4, 45, 46].

#### Scientific versus political processes of weighing up evidence and other priorities

The rationalist model of evidence-based policy-making assumes a linear process from evidence production—favouring randomized controlled designs and systematic reviews of such trials according to the prevailing heuristic of the evidence hierarchy—to its direct uptake in the policy-making process [47]. However, a view on political decision-making as a rather technical search for solutions to any given problem falls short of the realities of decision-makers who negotiate what is socially desirable in complex systems of governance. The remarkable successes in health care following the widespread adoption of the principles and methods of evidence-based medicine led to attempts by scholars and practitioners to similarly increase the use of scientific evidence in other areas and in policy-making at large [4]. It was purported that controlled experiments and evidence syntheses—given their pivotal role in helping to effectively address a range of medical issues—would be equally well-suited to improve political decision-making in other areas such as social policy. However, as with clinical studies where statistical significance does not always translate to medical relevance, proven effectiveness of any policy does not necessarily translate into social importance, let alone societal support for implementation of that policy. The consideration of patient values is therefore—at least in principle—an integral part of practising evidence-based medicine. Similarly, normative concerns around local needs and social norms and values are as, if not more, important in influencing decision-making as is scientific evidence. Relatedly, lack of implementation features and of context-specificity were pointed out by participants as potentially limiting the S3-guideline’s impact on decision-making. This appears to be in line with previous findings on the limited impact of guidelines in decision- and policy-making [39, 48]. The WHO-INTEGRATE Evidence-to-Decision framework was applied as part of the guideline development process to assess potential (unintended) effects of measures beyond health outcomes transparently and systematically, considering both evidence and expertise, including experiential expertise [20]. Even though this approach was intended to cater towards and mirror the complex realities of political decision-making, the guideline was mostly not perceived to have played a significant role in decision-making processes. Participants saw the guideline’s value in its systematic, transparent and consensual development process but also judged recommendations that were agnostic to local context or not grounded in most recent evidence as less attractive or useful to decision-makers. Similarly, the guideline does not seem to have played a guiding role for the school family in their respective contexts; it did, however, present a source of reassurance for some. Lack of impact of the guideline might in part be due to a missing in-depth understanding of the methodological approach taken to arrive at recommendations among decision-makers. It might also reflect limitations to the extent to which an independent process of weighing up different aspects, including evidence of effectiveness, can inform or cut short similar processes of negotiating societal priorities in the political realm. Both assumptions would have to be scrutinized in future research.

#### Utilization of the guideline in decision-making processes

This guideline was a novel instrument in the German (emergency) public health decision-making context. Hence, whether, how, why and by whom it would be used was largely unpredictable. Generally, the way in which scientific evidence of whatever discipline or format is taken up in decision-making can vary. It can be *instrumental*, i.e., use of evidence to solve a particular problem; *conceptual*, i.e., use of evidence to understand a particular phenomenon or *symbolic*, i.e., use of evidence to legitimise predetermined or retroactively justify past decisions [49]. Our findings suggest various ways of how the guideline was used: in some Federal States, it was probably used symbolically to confirm existing directives. In other Federal States, the guideline appears to have been used instrumentally or conceptually to inform decisions. It has been argued that the idea of instrumental use reflects “rather unrealistic assumptions about who is involved, what they represent, and the best way to make policy” [50] and that”it probably takes an extraordinary concatenation of circumstances for research to influence policy decisions directly” [49].

#### Improving evidence-advisory systems for public health decision-making in Germany

In Germany, the AWMF has been mandated to develop guidelines to provide guardrails for and improve the outcomes of clinical practice [51]. The S3-guideline for controlling SARS-CoV-2 transmission in German schools was developed according to the methodological standards established by the AWMF but targeted political and practical decision-makers rather than clinical practitioners, based on the assumption that this established process can be transferred from the health care to the policy and school realm. To achieve “good governance of evidence” [4], i.e. legitimacy and the use of “appropriate evidence of high quality” (ibid) in negotiating societal interests in Germany, critical analysis of the prevailing evidence-to-policy institutional arrangements is warranted. It needs to be established whether and what type of evidence-advisory institutional arrangements are needed to successfully inform public health (emergency) policy-making in Germany. Examples from other countries such as the British National Institute for Health and Care Excellence [52]—which has a political mandate to provide evidence syntheses and recommendations but does not hold decision-making power—could inform such a process.

### Methodological strengths and limitations of this study

Due to lack of dedicated funding, an external evaluation of the guideline’s impact was not feasible. We aimed to minimise any biases in the design and execution of the study, namely, data collection and analysis were solely carried out by KW and MR who were not involved with guideline development.

To keep the evaluation feasible, we decided to recruit in two Federal States only, thus limiting the representativeness of our findings. In light of the challenges with recruitment of individuals involved in decision-making (see below) and to further integrate the findings from the FoIA, extending recruitment to the remaining Federal States would have been warranted. However, time and resource constraints made this impossible. We cannot infer from the cross-sectional findings in component 1 whether the guideline played a role in informing updates of directives over time. Recruitment for component 2 was challenging, mostly due to major time constraints among decision-makers during an ongoing pandemic. Some potential interview participants also refused to participate, stating that they were not making decisions regarding school measures or felt they could not offer an individual perspective on these multifactorial and multi-actor processes. We could not recruit any individual with a political mandate. Diversity of professional and institutional background within the sample was low, given that four individuals represented two institutions in Bavaria and only one individual from a ministry could be recruited from Bremen. Hence saturation regarding the potential range of perspectives of those informing or making political decisions was likely not achieved.

During the interviews, participants explicitly stated that they could not and/or did not want to provide details about the decision-making processes regarding school measures within the concerned ministries. To enable a conversation that would be as open as possible, we reiterated that we were not interested in any personal or otherwise compromising details but rather in developing a conceptual understanding of the processes of informing and arriving at decisions regarding school measures. We also confirmed that all manuscripts would be fully anonymised and that potentially compromising statements would not be included in any publications. We are confident that participants trusted these assertions as they described their insights in much detail. As participants were interviewed about a period of almost 1 year since the release of the first short version of the guideline, recall bias might have been present.

During data analysis, the development of main and sub-categories was undertaken by one researcher (KW) only. Intra-coder reliability was established by critically examining the category system repeatedly. The results were validated by MR. The data created with individuals involved in decision-making was moderately rich. This was likely due to participants’ duty of confidentiality regarding political processes or lack of insight into these. More explicit grounding of this study in political theory and systems thinking from the design phase onwards might have enabled us to create richer data in these interviews. To present our findings to an international audience, KW translated quotes as well as further material such as the FoIA inquiries from German into English, which were then checked by a second author (MR). This might have caused some loss or change of meaning.

A process of reflecting about the researchers’ positionality accompanied the full research process and the interpretation of findings was scrutinised by all authors through repeated discussion. Integration with the responses to FoIA inquiries could only be carried out narratively given that no responses to the FoIA inquiries were available from Bavaria and Bremen. Hence, our findings regarding the impact of the S3-guideline are indicative rather than conclusive.

### Implications for policy and practice, and for research

Calls for better pandemic preparedness have been paramount since the start of the COVID-19 pandemic [53–55]. This could include putting in place the organisational and financial conditions for the continuous collection and assessment of evidence and expertise from the onset of a potential health crisis. Timely access to good quality and relevant research evidence, collaborations with decision-makers and relationship- and skills-building with decision-makers have previously been reported to be important factors in influencing the use of evidence [56]. Beyond training decision-makers in scientific thinking, efforts might be needed to enhance mutual recognition of the limits to scientific evidence and to evidence-based policy-making by scientists and decision-makers alike [4, 46].

Previous research on the uptake of clinical guidelines has suggested that including information on how to implement measures recommended in guidelines could enhance their uptake and use [57–59]. It was also found that in-house expertise is usually a trusted source of evidence for decision-makers, which was also reflected in our findings [60]. Further elements to enhance guideline uptake previously suggested by researchers include details regarding further implications of implementation such as costs or human and technical resources [57]. Guidance as to how to adapt national-level guidelines to match specific needs in a context-sensitive way might also prove to be useful [58]. This suggests a need for guideline panels to include expertise and evidence from fields such as economics, law, and organisational management to increase utility.

The implementation of public health guidelines is rarely comprehensively evaluated [41]. In light of the considerable resources taken up by guideline development processes, both under normal and pandemic circumstances, it is important to assess the usability, utility and actual use of the output. But examining decision-making processes exclusively with regards to whether, how much, or how quickly evidence (or evidence-based tools such as the S3-guideline for that matter) are taken up can fall short of the complexity inherent to decision-making. Therefore, evaluators should take a critical stance towards overly simplistic conceptualisations of evidence use in policy. Implementation research on the use of guidelines should be grounded in systems thinking and informed by political theory. A combination of different methods and triangulation of results may be appropriate. This may include nationally representative surveys, interview studies with individuals involved in political and practical decision-making, ethnographic research, media analysis as well as policy document analysis.

Guideline development processes may need to be adapted to account better for the needs of political and practical decision-makers and the realities of policy-making, and to integrate diverse types of evidence to enhance their usability, utility and use for public health (emergency) decision-making. We suggest that further work, both theoretical as well as dialogical with research, policy and practice actors, is needed to establish whether evidence- and consensus-based guidelines represent “good evidence” [4] for policy-making under uncertainty at all and what “good use of [this type of] evidence” [4] would require in terms of institutionalisation of evidence-advisory systems, in Germany and internationally.

## Conclusions

Our findings suggest that the S3-guideline was relatively well-known in Federal States’ decision-making bodies and that it was considered alongside other forms of evidence in some of these. More attention to the complex dynamics of and competing influences on political and practical decision-making processes could help assess whether public health guidelines are appropriate tools for policy advice during crises and beyond. Further research to evaluate the impact of public health guidelines on political and practical decision-making at national as well as Federal State levels is warranted, and can and should make use of a broad range of scientific approaches. Public health guideline development processes may need to be adapted to account for the realities of decision-making, during public health emergencies and during regular times. Lastly, such processes to inform public health policy-making might require a wider process of forming systems for the good governance of evidence, both in Germany and internationally.

## Data Availability

To maintain confidentiality, original data is only available upon request.

## Appendices

### Appendix 1: Example inquiry letter according to Freedom of Information Acts in English and German

#### English

Dear Sir or Madam,

We are researchers at the Ludwig-Maximilians-Universität of Munich in the Department of Public Health and Health Services Research.

Over the past few months, we have been conducting research on the topic of schools in the SARS-CoV-2 pandemic. Our focus was on the extent to which the measures implemented in schools to prevent and control the pandemic are effective. The results of this research were used in the development of an evidence- and consensus-based S3-guideline. (https://www.awmf.org/leitlinien/detail/ll/027-076.html).

The guideline development process was coordinated by us, and a large number of professional societies, institutions and associations were involved in its preparation. The aim of the guideline is to provide decision-makers in the fields of education and health with scientifically sound and consensual recommendations for action in order to enable the safest, most regulated and continuous school operation possible in times of pandemic.

The abridged version of the guideline was published on 8 February 2021. It is not yet clear to what extent the recommendations of the guideline will be implemented in the individual Federal States. With reference to the Freedom of Information Act, we therefore request the following information:

1. Is the Ministry aware of the guideline?
  - How did the Ministry become aware of the guideline?
  - To whom was the guideline further distributed—within the ministry and beyond?
2. Has the guideline been discussed in the ministry?
  - If so, in which department/unit and/or in which committee/task force and/or at which hierarchical level was the guideline discussed?
  - If yes, which aspects of the guideline were discussed?
3. Was the guideline taken into account in decisions in the ministry or in decisions in which the ministry was involved?
  - If yes, in which decisions was the guideline taken into account?
  - If yes, which recommendations from the guideline were taken into account and how?
  - If no, why was the guideline not taken into account?
4. Has the guideline had a concrete impact on ministerial advice, recommendations or guidelines for schools?
  - If yes, what specific changes (e.g. regulation on masks, regulation on routes to school) have resulted from the guideline?
  - If you are not responsible for this question, please forward this question to the competent authority.

We request an answer in electronic form (e-mail to leitlinie@ibe.med.uni-muenchen.de) as well as an acknowledgement of receipt.

Please do not hesitate to contact us if you have any questions.

Yours sincerely.

#### German

Sehr geehrte Damen und Herren,

wir sind als Wissenschaftler:innen an der Ludwig-Maximilians-Universität München am Lehrstuhl für Public Health und Versorgungsforschung tätig.

Während der vergangenen Monate haben wir zum Thema Schulen in der SARS-CoV-2 Pandemie geforscht. Im Zentrum stand die Frage, wie wirksam die Maßnahmen sind, die an Schulen zur Prävention und Kontrolle der Pandemie umgesetzt wurden. Die Ergebnisse dieser Forschung wurden bei der Entwicklung einer evidenz- und konsensbasierten S3-Leitlinie genutzt (https://www.awmf.org/leitlinien/detail/ll/027-076.html).

Der Leitlinienprozess wurde von uns koordiniert, an der Erstellung waren eine Vielzahl von Fachgesellschaften, Institutionen und Verbänden beteiligt. Ziel der Leitlinie ist es, Entscheidungsträger:innen in den Bereichen Bildung und Gesundheit wissenschaftlich fundierte und konsentierte Handlungsempfehlungen zur Verfügung zu stellen, um einen möglichst sicheren, geregelten und kontinuierlichen Schulbetrieb in Pandemiezeiten zu ermöglichen.

Die Kurzfassung der Leitlinie wurde am 8. Februar 2021 veröffentlicht. Inwieweit die Empfehlungen der Leitlinie in den einzelnen Bundesländern umgesetzt werden ist bisher unklar. In Berufung auf das Informationsfreiheitsgesetz erbitten wir daher folgende Informationen:

1. Ist die Leitlinie im Ministerium bekannt?
  - Wie wurde das Ministerium auf die Leitlinie aufmerksam?
  - An wen wurde die Leitlinie – innerhalb des Ministeriums und darüber hinaus – weiter verteilt?
2. Wurde die Leitlinie im Ministerium diskutiert?
  - Wenn ja, in welcher Abteilung/Referat und/oder in welchem Gremium/Task Force und/oder auf welcher Hierarchieebene wurde die Leitlinie diskutiert?
  - Wenn ja, welche Aspekte der Leitlinie wurden diskutiert?
3. Wurde die Leitlinie bei Entscheidungen im Ministerium oder in Entscheidungen, an denen das Ministerium mitgewirkt hat, berücksichtigt?
  - Wenn ja, bei welchen Entscheidungen wurde die Leitlinie berücksichtigt?
  - Wenn ja, welche Empfehlungen aus der Leitlinie wurden berücksichtigt und wie?
  - Wenn nein, warum wurde die Leitlinie nicht berücksichtigt?
4. Hat sich die Leitlinie konkret auf ministerielle Hinweise, Empfehlungen oder Vorgaben für Schulen ausgewirkt?
  - Wenn ja, welche konkreten Änderungen (z.B. Regelung zu Masken, Regelung zu Schulwegen) haben sich aus der Leitlinie ergeben?
  - Falls Sie für diese Anfrage nicht zuständig sind, bitten wir Sie um Weiterleitung dieser Anfrage an die zuständige Behörde.

Wir bitten um eine Antwort in elektronischer Form (E-Mail an leitlinie@ibe.med.uni-muenchen.de), sowie um eine Empfangsbestätigung.

Für Rückfragen stehen wir gerne zur Verfügung.

Mit freundlichen Grüßen.

### Appendix 2: Interview guide in English and German.

#### English

**Introduction**

Please introduce yourself briefly with your name, function and institution.

Are you familiar with the guideline?

How did you learn about the guideline?

When did you learn about the guideline?

Were you involved yourself in the creation of guidance?

From whom or which institution did you receive guidelines or recommendations?

What is your role in the decision-making process regarding measures in schools?

How is your role defined in the pandemic? How does this differ from your "normal" work/function?

**Steps of and basis for decision-making processes**

How did and do the decisions on school measures come about in your Federal State?

Which institutions were and are involved and in which role?

If you were given guidance, how much leeway did you have to adapt it to your context?

To what extent have decisions at the national level influenced your decisions and actions?

How did and do decision-making processes regarding school measures take place in your institution?

To what extent was there a person/institution with ultimate decision-making power in the decision-making processes?

**Knowledge translation generally**

How do scientific findings find their way into politics?

Through which channels or which instruments do scientific findings find their way into decision-making processes in your institution?

**Role of the guideline in decision-making**

To what extent did the guideline play a role in the decision-making processes on school measures?

When did the guideline play a role?

**The guideline as an instrument for science-based policy advice**

In your view, how important is such a guideline in the context of political and practical decision-making during crises?

How important was the guideline for politicians and other decision-makers in your view?

How do you assess the role of such a guideline during political decision-making?

What can such a guideline achieve during a crisis?

What can such a guideline not do during a crisis?

To what extent have you worked with guidelines before?

**Understanding of evidence and expertise**

What do you understand by scientific evidence?

How is expertise to be understood in contrast to evidence in the context of such decision-making processes?

What value do you attach to evidence in the context of this and other decision-making processes?

**Closing**

Would you like to say anything else about the guideline or the decision-making processes?

Are there any aspects that have not been mentioned so far but which you think could play a role?

Do you think we have forgotten to mention anything?

#### German

**Einleitung**

Bitte stellen Sie sich kurz mit Ihrem Namen, Ihrer Funktion und Ihrer Institution vor.

Sind Sie mit der Leitlinie vertraut?

Wie haben Sie von der Leitlinie erfahren?

Wann haben Sie von der Leitlinie erfahren?

Waren Sie selbst an der Erstellung von Vorgaben beteiligt?

Von wem bzw. welcher Institution haben Sie Vorgaben oder Empfehlungen erhalten?

Welche Rolle spielen Sie bei der Entscheidungsfindung über Maßnahmen in Schulen?

Wie ist Ihre Rolle in der Pandemie definiert? Wie unterscheidet sich diese von Ihrer "normalen" Arbeit/Funktion?

**Ablauf und Grundlagen der Entscheidungsprozesse**

Wie kamen und kommen die Entscheidungen bzgl der Schulmaßnahmen in Ihrem Bundesland zustande?

Welche Institutionen waren und sind in welcher Rolle beteiligt?

Wenn Ihnen Vorgaben gemacht wurden, wie viel Spielraum hatten Sie, diese an Ihren Kontext anzupassen?

Inwieweit haben Entscheidungen auf nationaler Ebene Ihre Entscheidungen und Handlungen beeinflusst?

Wie liefen und laufen Entscheidungsprozesse bzgl. der Schulmaßnahmen in Ihrer Einrichtung ab?

Inwieweit gab es bei den Entscheidungsprozessen eine Person/Institution mit letzter Entscheidungsgewalt?

**Wissenstransfer allgemein**

Wie finden wissenschaftliche Erkenntnisse ihren Weg in die Politik?

Über welche Kanäle bzw. welche Instrumente finden wissenschaftliche Erkenntnisse Eingang in die Entscheidungsfindungsprozesse Ihrer Institution?

**Rolle der Leitlinie in Entscheidungsprozessen**

Inwieweit hat die Leitlinie bei den Entscheidungsprozessen zu Schulmaßnahmen eine Rolle gespielt?

Wann hat die Leitlinie eine Rolle gespielt?

**Die Leitlinie als Instrument zur wissenschaftsbasierten Politikberatung**

Wie wichtig ist Ihrer Meinung nach eine solche Leitlinie im Rahmen der politischen und praktischen Entscheidungsfindung in Krisenzeiten?

Wie wichtig war die Leitlinie Ihrer Meinung nach für Politiker:innen und andere Entscheidungsträger:innen?

Wie beurteilen Sie die Rolle einer solchen Leitlinie bei der politischen Entscheidungsfindung?

Was kann eine solche Leitlinie in einer Krise leisten?

Was kann eine solche Leitlinie in einer Krise nicht leisten?

Inwieweit haben Sie vorher schon mit Leitlinien gearbeitet?

**Verständnis von Evidenz und Expertise**

Was verstehen Sie unter wissenschaftlicher Evidenz?

Wie ist Expertise im Gegensatz zu Evidenz im Zusammenhang mit solchen Entscheidungsprozessen zu verstehen?

Welchen Stellenwert messen Sie der Evidenz im Rahmen dieser und anderer Entscheidungsprozesse bei?

**Schlusswort**

Möchten Sie noch etwas zur Leitlinie oder zu den Entscheidungsprozessen sagen?

Gibt es noch Aspekte, die bisher nicht zur Sprache kamen, Ihrer Meinung aber eine Rolle spielen könnten?

Haben wir Ihrer Meinung nach etwas vergessen abzufragen?

### Appendix 3: Deductively developed main categories with inductively developed sub-categories

**Table:**

Code system	Memo
The impact of the S3-guideline on political and practical decision-making regarding school measures	Includes statements regarding the influence on practical decision-making and implementation processes that the guideline (and guidelines in general) has had or may have had
Value of the guideline for decision-making	
The role of the S3-guideline in political decision-making	
The role of the S3-guideline for decision-making in schools	
Limitations to the guideline’s impact	
The impact of the S3-guideline on political decision-making processes regarding school measures	Includes statements on the role of the S3-guideline in the decision-making process within the political institutions responsible for school measures in the respective Federal State, as well as on the role of guidelines in general in decision-making processes
The role of the S3-guideline in informing political decisions	
The role of the S3-guideline in making political decisions	
Procedures of decision-making regarding school measures	Includes the process of decision-making within the political institutions responsible for measures in schools in the respective Federal State
Hierarchies in the development and implementation of decisions	
Sources and channels of information and advice for decision-makers	
Consideration of unintended consequences and further aspects when informing and making decisions	

### Appendix 4: Tabular overview of results in component 1

**Table:**

Federal State	a) Is the guideline known in the ministry for education?	b) Was the guideline discussed in the ministry for education?	c) Was the guideline considered for decisions?	d) Did the guideline influence information, recommendations, or requirements for schools?	Illustrative quotes from response letters
Baden-Württemberg	Yes	Unclear	Yes	Yes	
Bavaria	No answer	No answer	No answer	No answer	
Berlin	No answer	No answer	No answer	No answer	
Brandenburg	No answer	No answer	No answer	No answer	
Bremen	Yes	No answer	No answer	No answer	
Hamburg	Unclear	Unclear	Unclear	Unclear	*“In light of this development, the publication of the S3-guideline in February 2021 happened quite late. Many of the measures mentioned there were already implemented in Hamburg in 2020. The BSB [Hamburg Education Authority, Behörde für Schule und Berufsbildung Hamburg] coordinates the measures for the school sector with the health authority where recommendations […] such as relevant guidelines are evaluated […] Whether the S3 guideline is known in the BSB's area of responsibility, and if so, to what level of detail, is not known.”*
Hesse	Yes	Yes	Yes	Yes	
Mecklenburg-West Pomerania	Yes	Yes	Yes	Yes	*“A detailed check against the guideline was carried out […] with regards to the infection protection and hygiene measures in force at schools at the time in question […] The […] examination concluded that the majority of recommendations are already […] being implemented and that there is […] no increased need for action […]. …] With regard to physical education which did not take place due to the general school closures at the beginning of 2021, the S3-guideline contributed to a regulation in the hygiene plan for the schools, according to which physical education can be carried out in those grades that are taught in presence within the framework of the school's organisational discretion.”*
Lower Saxony	No answer	No answer	No answer	No answer	
North Rhine-Westphalia	No answer	No answer	No answer	No answer	
Rhineland-Palatinate	Yes	Yes	Unclear	Unclear	
Saarland	Yes	Yes	Yes	Yes	*“We were able to determine that we were largely in line with the recommendations of the S3-guideline with our specifications on hygiene and infection protection at schools. Only regarding the obligation to wear masks, we "only" recommended face coverings and not medical masks as recommended in the guideline.”*
Saxony	Yes	Yes	Yes	Unclear	*“The guideline is one of many pieces of information to be considered. No detailed information can be given retroactively as to which specific information is to be found where in the [hygiene framework directive for schools of Federal State M]. This would have warranted a separate request when the guideline was published.”*
Saxony-Anhalt	Yes	Yes	Yes	Unclear	*“The guideline was and is being considered in the ongoing revision of the [hygiene framework directive for schools of Federal State N]. It was found that most recommendations formulated in the S3-guideline had been implemented […] the guideline confirmed the hygiene measures already taken in schools by [Federal State N].”*
Schleswig–Holstein	Yes	Yes	Yes	Yes	
Thuringia	No answer	No answer	No answer	No answer	

## Abbreviations

BSB: Hamburg school authority (Behörde für Schule und Berufsbildung)
PSHM: Public health and social measures
COVID-19: Coronavirus disease 2019
SARS-CoV-2: Severe acute respiratory syndrome coronavirus 2
School family: Students, parents and teachers
School measures: Measures to keep schools open safely by preventing and controlling transmission of SARS-CoV-2
RKI: Robert Koch Institute
FoIA: Freedom of Information Acts
COREQ: Consolidated criteria for reporting qualitative research
STIKO: Standing Committee on Vaccination
AWMF: Association of the Scientific Medical Societies in Germany
Ministries: Federal State ministries or departments

## Footnotes

1 Clinical guidelines by the Association of the Scientific Medical Societies in Germany (Arbeitsgemeinschaft für Medizinisch-Wissenschaftliche Fachgesellschaften, AWMF) are classified according to the extent to which elements of systematic development are applied. The label “S3” requires systematic searches for published scientific evidence and an assessment of the quality of this evidence, as well as structured consensus-building and the representation of stakeholders on the panel. It constitutes the highest level of guidelines according to this classification.
