# Correction: Assessing the impact of an evidence- and consensus-based guideline for controlling SARS-CoV-2 transmission in German schools on decision-making processes: a multi-component qualitative analysis

**DOI:** 10.1186/s12961-025-01346-4

**Published:** 2025-06-02

**Authors:** Katharina Wabnitz, Mike Rueb, Eva A. Rehfuess, Brigitte Strahwald, Lisa M. Pfadenhauer

**Affiliations:** 1 Institute for Medical Information Processing, Biometry and Epidemiology (IBE), Chair of Public Health and Health Services Research, Ludwig-Maximilians Universität München, Elisabeth-Winterhalter-Weg 6, 81377 Munich, Germany; 2 Pettenkofer School of Public Health, Munich, Germany

**Correction: Health Research Policy and Systems (2023) 21:138 ** 10.1186/s12961-023-01072-9

Following publication of the original article [1] it was reported that there was an error in Fig. 1c, Fig. 1d and in Appendix 4.

In Fig. 1c Thuringia was wrongly coded in green and should have been grey.

Figure 1d indicated four Federal States classified as having reported that the guideline influenced recommendations or guidelines for schools (in green), but this should be five.

In the Table of Appendix 4 the response given in column ‘d) Did the guideline influence information, recommendations, or requirements for schools?’ for the Federal State Hesse was given as ‘Unclear’ but should be ‘Yes’.

The incorrect and correct versions of Fig. 1 and the corrected version of Appendix 4 with the corrected response in bold are given below. The original article has been updated.

Incorrect Fig. 1
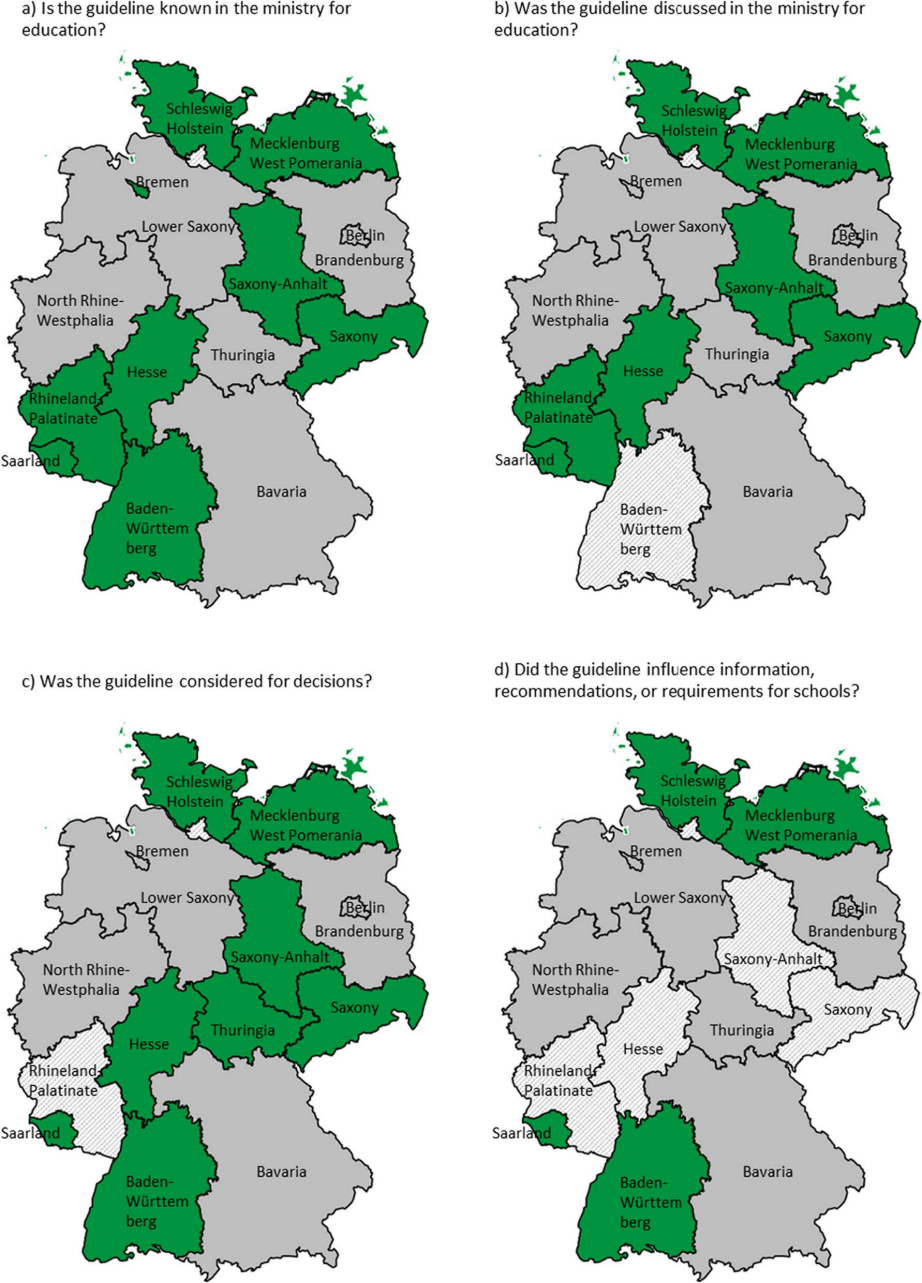
 .

Correct Fig. 1

**Fig. 1:**
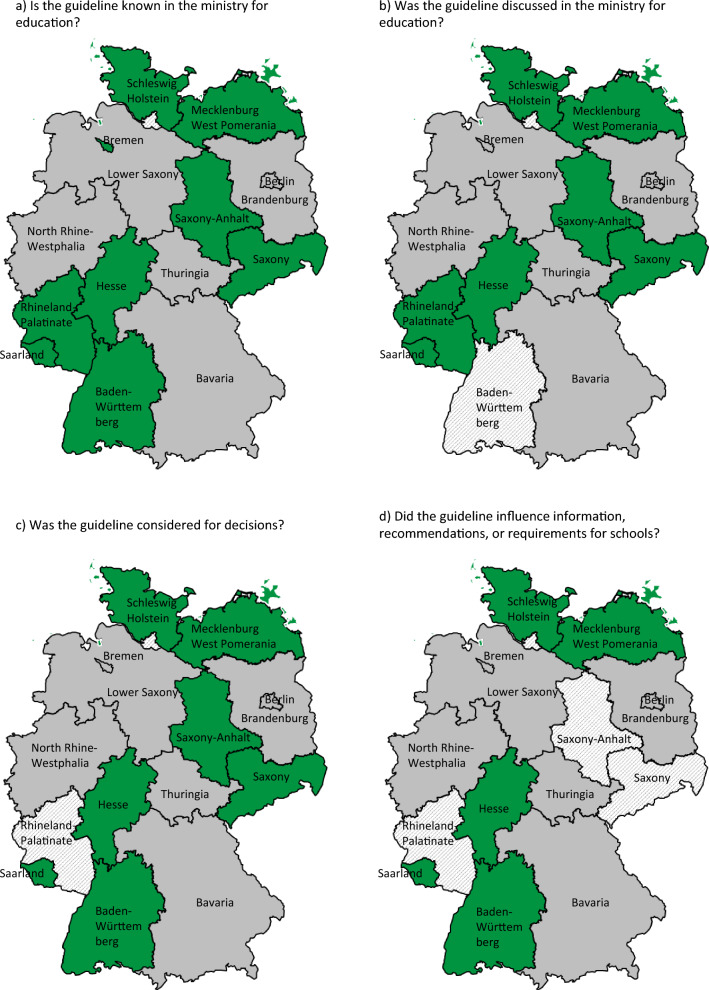
Impact of the S3-guideline on decision-making processes, according to answers to four main questions as part of the FoIA inquiries by Federal States. Green = yes, red = no, grey = no answer, shaded = unclear

.

Correct Appendix 4.

Annex 4: Tabular overview of results in component 1.

**Table:** 

Federal State	a) Is the guideline known in the ministry for education?	b) Was the guideline discussed in the ministry for education?	c) Was the guideline considered for decisions?	d) Did the guideline influence information, recommendations, or requirements for schools?	Illustrative quotes from response letters
Baden-Württemberg	Yes	Unclear	Yes	Yes	
Bavaria	No answer	No answer	No answer	No answer	
Berlin	No answer	No answer	No answer	No answer	
Brandenburg	No answer	No answer	No answer	No answer	
Bremen	Yes	No answer	No answer	No answer	
Hamburg	Unclear	Unclear	Unclear	Unclear	*“In light of this development, the publication of the S3-guideline in February 2021 happened quite late. Many of the measures mentioned there were already implemented in Hamburg in 2020. The BSB [Hamburg Education Authority, Behörde für Schule und Berufsbildung Hamburg] coordinates the measures for the school sector with the health authority where recommendations […] such as relevant guidelines are evaluated […] Whether the S3 guideline is known in the BSB's area of responsibility, and if so, to what level of detail, is not known.”*
Hesse	Yes	Yes	Yes	**Yes**	
Mecklenburg-West Pomerania	Yes	Yes	Yes	Yes	*“A detailed check against the guideline was carried out […] with regards to the infection protection and hygiene measures in force at schools at the time in question […] The […] examination concluded that the majority of recommendations are already […] being implemented and that there is […] no increased need for action […]. …] With regard to physical education which did not take place due to the general school closures at the beginning of 2021, the S3-guideline contributed to a regulation in the hygiene plan for the schools, according to which physical education can be carried out in those grades that are taught in presence within the framework of the school's organisational discretion.”*
Lower Saxony	No answer	No answer	No answer	No answer	
North Rhine-Westphalia	No answer	No answer	No answer	No answer	
Rhineland-Palatinate	Yes	Yes	Unclear	Unclear	
Saarland	Yes	Yes	Yes	Yes	*“We were able to determine that we were largely in line with the recommendations of the S3-guideline with our specifications on hygiene and infection protection at schools. Only regarding the obligation to wear masks, we "only" recommended face coverings and not medical masks as recommended in the guideline.”*
Saxony	Yes	Yes	Yes	Unclear	*“The guideline is one of many pieces of information to be considered. No detailed information can be given retroactively as to which specific information is to be found where in the [hygiene framework directive for schools of Saxony]. This would have warranted a separate request when the guideline was published.”*
Saxony-Anhalt	Yes	Yes	Yes	Unclear	*“The guideline was and is being considered in the ongoing revision of the [hygiene framework directive for schools of Saxony-Anhalt]. It was found that most recommendations formulated in the S3-guideline had been implemented […] the guideline confirmed the hygiene measures already taken in schools by Saxony-Anhalt.”*
Schleswig–Holstein	Yes	Yes	Yes	Yes	
Thuringia	No answer	No answer	No answer	No answer	
